# Scale-up of hepatic progenitor cells from multitray stack to 2-D bioreactors

**DOI:** 10.1186/1753-6561-7-S6-P61

**Published:** 2013-12-04

**Authors:** Matthieu Egloff, Florence Collignon, Jean-François Michiels, Jonathan Goffinet, Sarah Snykers, Philippe Willemsen, Christophe Gumy, Claude Dedry, Jose Castillo, Jean-Christophe Drugmand

**Affiliations:** 1 ATMI LifeSciences, Brussels, Belgium, 1120; 2 Promethera Biosciences, Mont-Saint-Guibert, 1435, Belgium

## Introduction

Promethera Biosciences (Mont-St-Guibert, BE) is developing cell therapies to treat several liver genetic metabolic diseases, such as the Crigler-Najjar syndrome. Human heterologous adult liver progenitors cells (HHALPCs) were initially cultivated in 2D standard cultivation devices. The present study is investigating the feasibility to cultivate HHALPCs in Xpansion bioreactors, with the following objectives:

➢ The process must be closed

➢ The growth rate and population-doubling level (*i.e*. the number of times the cells in the population has doubled) must be at least equivalent to the current process in multilayer trays

➢ The process must comply to the cGMP rules

➢ The cells must succeed the quality control (QC) test specifications at the end of cultivation, *i.e*. cells must remain undifferentiated and show the presence of HHAPLCs markers, while exhibiting the capacity to differentiate toward functional hepatocytes.

Integrity^® ^Xpansion™ multiplate bioreactors have been specifically designed to enable an easy transfer from existing multiple-tray-stack processes by offering the same cell growth environment on 2-D hydrophylized Polystyrene (PS) plates in a fully closed system. To make the bioreactors compact, the headspace between each plate has been reduced to a minimum (1.3 mm). Gas transfer is made through a semi-permeable silicone tubing mounted in the central column. Additionally, critical cell culture parameters such as pH and DO are controlled and the cell density is automatically monitored via a specific holographic microscope developed by Ovizio

## Materials and methods

### Cell culture parameters

✓ pH set-point: 7.5

✓ DO regulated > 50%

✓ No agitation during the first 8 hours after plating

### Stem cells expansion and harvesting

✓ Inoculation: 5,000 cells/cm^2^

✓ Harvest: 20,000-40,000 cells/cm^2^

✓ 10% serum-containing medium

## Results

Xpansion 10 was used to prove feasibility of stem cell growth in Xpansion multiplate bioreactor and to optimize cell culture parameters. The goal was to perform a simple process transfer from multitray stack (e.g. Corning CellStack (CS)) to the Xpansion by mimicking cell culture conditions.

All Xpansion runs achieved similar results to their control in terms of cell density, homogenous distribution, viability and morphology. Additional quality control (QC) analysis revealed that cell characteristics were maintained (identity/purity/potency) (table 1)

**Table 1:** Scale-up feasibility of stem cells growth in Xpansion bioreactor.

QUALITY CONTROL TEST	Xpanion10 (Five runs)	Xpansion 50 (Two runs)	Xpansion 180 (Three runs)	In-Line Centrifugation Three runs
CELL CULTURE SURFACE (CM^2^)	6.120	30.600	110.160	/
AVERAGE CELL QUANTITY AT HARVEST	1.8 × 10^8^	9 × 10^8^	3.3 × 10^9^	/
VIABILITY	≥90%	≥90%	≥90%	≥90%
GROWTH PROFILE	Normal	Normal	Normal	Normal
CONFLUENCY	√	√	√	√
HOMOGENEOUS CELL DISTRIBUTION & MORPHOLOGY	**√**	**√**	**√**	**√**
IDENTITY CYP3A4 Activity	**Conform** > 10^-8^pmol/cell/4 h	**Conform** > 10^-8^pmol/cell/4 h	**Conform** > 10^-8^pmol/cell/4 h	**Conform** > 10^-8^pmol/cell/4 h
IDENTITY Phenotype	**Conform** CD73, CD90>60% ALB+, vim+, ASMA+	**Conform** CD73, CD90>60% ALB+, vim+, ASMA+	**Conform** CD73, CD90>60% ALB+, vim+, ASMA+	**Conform** CD73, CD90>60% ALB+, vim+, ASMA+
PURITY	**Conform** CD31+CD133+ CD45+CK19 < 15%	**Conform** CD31+CD133+ CD45+CK19 < 15%	**Conform** CD31+CD133+CD45+CK19 < 15%	**Conform** CD31+CD133+CD45+CK19 < 15%
POTENCY (Urea secretion)	**ConforM** 4/5*	**ConforM** 1/2*	**Conform** **3/3**	**Conform** **3/3**
POTENCY (Bilirubin Conjugation)	**Conform** 4/5*	**Conform** **2/2**	**Conform** **(pending)**	**Conform** **(pending)**

Cell properties are checked throughout the scale-up process and results are expressed in terms of cell viability, confluence, morphology, growth and cell characterization (identity/purity/potency). * 1 QC failed in the Xpansion 10 & Xpansion 50 bioreactors but QC were similar to their respective CS control (not related to the bioreactor).

### Scale-up from the Xpansion 10 to the Xpansion 180

Cultures were directly transferred from the Xpansion 10 bioreactor to the larger scales Xpansion 50 and Xpansion 180 bioreactors, where cells reached similar levels of growth and confluence (Table 1). Further analysis of the cultures at all scales showed compliancy with the QC specifications. In order to keep the process within a closed system, cells harvested from Xpansion 180 were directly centrifuges. The in-line continuous centrifugation step achieved 80% yields while maintaining cells characteristics (Table 1).

### Xpansion bioreactor regulation

**Figure 1:**
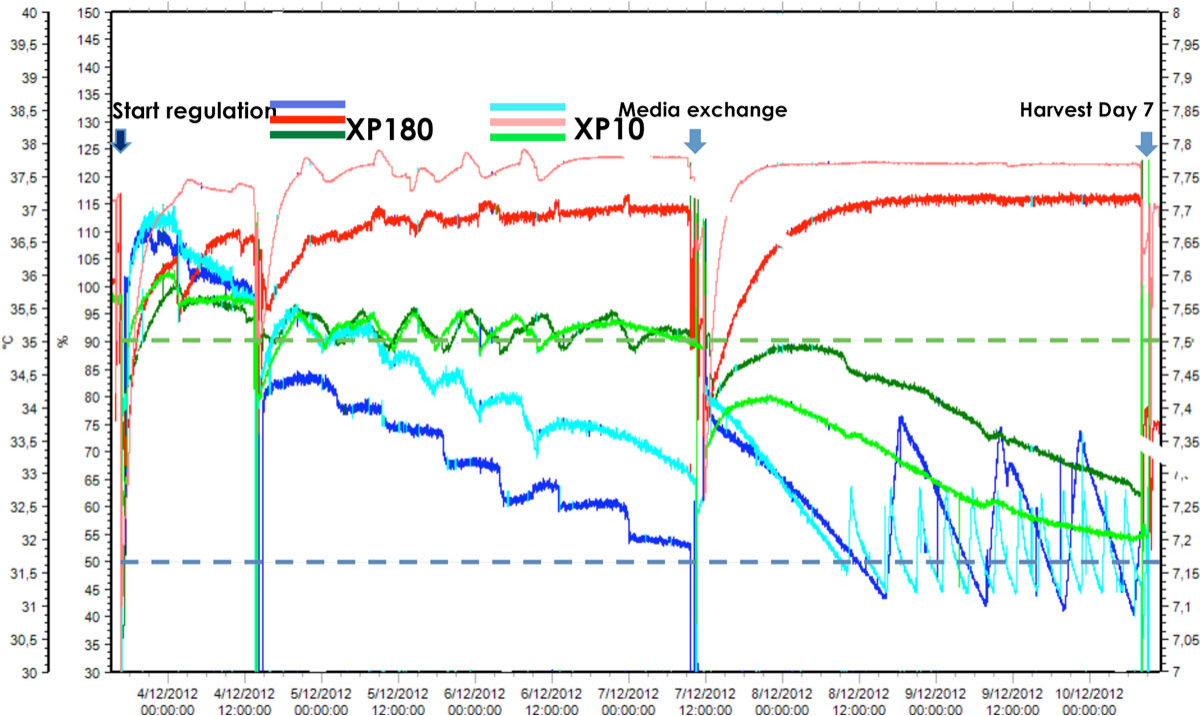
**Regulation parameters in XP-10 (A) or XP-180 (B) in the course of time, pH (green), D.O. (blue) and T° (red) evolution**. Set points (dashed lines) were fixed at 7.5 for pH and D.O. >50%. T° peaks are due to Xpansion disconnection for microscopic observation or samplings.

Figure 1 shows the pH and DO regulation profiles of cultures in Xpansion 10 and Xpansion 180. The trends of both bioreactors are highly similar, except that the duration of a regulation cycle is longer in the Xpansion 180 compared to the Xpansion10. This is due to the longer homogenization time. The gas diffusion system through the silicone tubing is efficient.

### Cell observation using the holographic microscope - iLine

The iLine holographic microscope and the Xpansion bioreactors are designed to allow cell observation on the first ten plates of each bioreactor. The microscope software enables an automatic cell counting of the cell confluency. Cell confluence assessment through DDHM microscope is a key element for defining cell harvest time given that cell confluence levels are critical to guarantee cell characteristics.

## Conclusions

The Integrity Xansion multiplate bioreactors demonstrated their efficiency for the growth of progenitor of hepatocyte cells at large scale while keeping the cell therapeutic potency.

The use of a robust process control system and the iLine microscope enabled to record the evolution of the culture:

➢ Sampling port that can be used for dosing of nutrients, growth factors, etc.

➢ On-line pH and D.O. tracking

➢ Off-line microscopic observations

The Xpansion 10 bioreactor proved to be a useful tool for determining optimal cell culture parameters. Actually, several runs could be performed using this scaled-down, while sparing time and money and extrapolating the cell behavior, the pH and DO trends in the Xpansion 50 and Xpansion 180. The new Xpansion bioreactor offers a valuable technology for large-scale production while meeting GMP compliancy. Moreover, the in-line centrifugation step guarantees a closed manufacturing process, from seeding to freezing.

